# Validation and Adaptation of the Academic-Resilience Scale in the Spanish Context

**DOI:** 10.3390/ijerph17113779

**Published:** 2020-05-26

**Authors:** Rubén Trigueros, Ana M. Magaz-González, Marta García-Tascón, Antonio Alias, José M. Aguilar-Parra

**Affiliations:** 1 Department of Language and Education, University of Antonio de Nebrija, 28015 Madrid, Spain; rtr088@ual.es; 2 Department of Education, University of Valladolid, 47002 Valladolid, Spain; anamaria.magaz@uva.es; 3 Faculty of Sports Science, University of Pablo de Olavide, 41704 Seville, Spain; margata@upo.es; 4 Department of Education, University of Almería, 04120 Almería, Spain; aag344@ual.es; 5 Department of Psychology, Hum-878 Research Team, Health Research Centre, University of Almería, 04120 Almería, Spain

**Keywords:** resilience, academic education, university students, factor analysis

## Abstract

The aim of this study was to validate and adapt the academic-resilience scale in the Spanish context. The study involved 2967 university students aged 18–33 (Mean, M = 23.65; Standard Deviation, SD = 2.57) from several universities in Andalusia (Spain). Exploratory and confirmatory factorial analyses revealed adequate adjustment rates for the new version of the scale showing the factorial structure invariant with respect to that generated. Three factors that integrate the scale obtained high correlation, internal consistency, and temporal stability. The Spanish version of the academic-resilience scale was shown to have adequate psychometric properties to measure academic resilience in the Spanish university context.

## 1. Introduction

Students throughout their academic period must, at some point, face multiple difficulties and tasks that place high cognitive and/or motor demands on them (e.g., test failure, friction with peers or teachers, and embarrassing situations) [1,2]. Therefore, the low capacity of students to adapt and overcome failure is one of the main indicators that can help understand early dropout from the education system, lack of promotion, and, in more serious cases, disruptive behavior in the classroom [3]. However, there are currently no instruments in Spanish with which to assess students’ academic resilience, and analyze whether they have sufficient defense mechanisms with which to adapt to the many difficulties they face on a daily basis. Therefore, this study aimed to adapt and validate the resilience-scale [4] questionnaire in the Spanish academic context.

The metatheory of resilience by Richardson [5] is one of the first theories that tries to explain how mechanisms available to people to try to overcome and adapt to the possible vicissitudes they face in their lives, and in their different work environments. The metatheory asserts that overcoming problems contributes to learning and personal progress since the individual responds to an adverse situation returning to a normal level of functioning [6]. Thus, the metatheory affirms that resilience begins in a situation of physical, mental, and spiritual balance that is disrupted when a situation arises in which the individual does not possess sufficient resources or skills to cope with adverse situations [5]. Over time, the individual readjusts and returns to balance by raising their level of resilience or homeostasis and acquiring the process skills and tools with which to overcome vicissitudes [5]. However, if the individual fails to reach equilibrium after a problem arises, their level of homeostasis decreases, and consequently their resilience [5]. In this sense, different studies showed how anxiety, depression, and stress contribute to the decrease of resilience due to continuous failure [7,8,9].

Despite the importance of the metatheory of resilience, recent studies in the field of sport [10] and education [11] showed that this theory has certain limitations. One of the limitations of metatheory is that it analyzes resilience as a construct from a linear point of view where an event causes a personal maladjustment in the individual, without considering other paths. Furthermore, it does not analyze how emotions can affect the process of adaptation and overcoming vicissitudes [12]. This is essential given the importance of emotions in protecting the individuals’ behaviors [13], and several studies showed that people value emotions as facilitators of developing resilience capacity [14]. These limitations led to the current reconceptualization of the notion of resilience as a construct associated with the presence of protective and vulnerable factors within and outside the individual that influence their adaptation to the problems that cause a break in homeostasis [15].

Research on resilience mainly focused on the fields of social psychology [16], the military [17], labor [18], medicine [19] and sports [20]; in the academic field, it is a construct that is still not present in various studies. These and other studies related to resilience showed a positive relationship towards coping with stressful events [21], quality of life [22], emotional regulation [23], emotional intelligence [24], and motivation [25]. Resilience, on the other hand, has been negatively associated with negative emotional states (e.g., anxiety, depression; [22]) and risk behaviors.

Multiple tools were used to assess resilience in the general population, such as the Connor–Davidson resilience scale [26] and the academic-resilience scale (ARS; [27]), although these tools were developed from rait methodology [5]. Another tool for assessing resilience that was developed and widely used is the resilience scale validated by Wagnild and Young [28]. This tool consists of two factors that provide insight into positive personality characteristics, personal competence (defined as recognition of personal capacity), independence, perseverance, and ability and acceptance of oneself and life as an equivalent to adaptation and flexibility. However, these instruments were originally created to measure resilience in social situations or to study the personality of the individual in the face of life’s vicissitudes; therefore, they have a conceptual bias that is less appropriate to the academic field. Cassidy [4] created the academic-resilience scale (ARS) to measure student resilience in the academic context. This instrument consists of 30 items distributed among three factors: perseverance, adaptation and help seeking, and negative affect and emotional response. A sample of 532 university students was used to validate the scale. In order to analyze the factorial structure of the questionnaire, exploratory factorial analysis was performed that confirmed the structure of the 30 items distributed among the three factors with a Kaiser–Meyer–Olkin of 0.897. In addition, it analyzed the predictive validity of the scale showing that the academic resilience of the university student positively predicted their academic self-efficacy.

Thus, the main objective of this study was to analyze the psychometric properties of the academic-resilience scale (ARS, [4]) in order to validate it and adapt it to the Spanish context. To this end, exploratory- and confirmatory-factor analyses, analysis of internal consistency, temporal stability, and gender-invariance analysis were performed. In addition, a second study is proposed that aims to analyze the scale’s predictability with respect to the academic stress of university students through a structural equation model (SEM). The hypothesis is that resilience negatively predicts students’ academic stress.

## 2. Materials and Methods

### 2.1. Participants

A total of 2967 university students (1461 men and 1506 women), aged between 18 and 33 (M = 23.65; SD = 2.57), from several universities in Andalusia (Spain) participated in this study. For exploratory factorial analysis, 565 university students between 18 and 30 years participated. For confirmatory factorial analysis, 723 university students between 18 and 33 years participated. For temporal stability analysis, 431 university students between 18 and 32 years took part. Finally, for criterion validity analysis, a sample of 1248 university students between the ages of 18 and 33 was used.

An incidental nonprobabilistic sampling was followed for the selection of participants on the basis of those students to whom we had access. The criteria for participation in the study was being a university student and the delivery of signed informed consent.

### 2.2. Procedure

In order to carry out the validation of the academic-resilience scale (ARS), an adaptation of the Cassidy academic-resilience scale was made [4]. The Hambleton procedure [29] was followed. This strategy consisted of a translation of the original scale into Spanish by a group of translators. Later, another group of translators translated the scale from Spanish into the original language of the scale. Each of the translators had more than 10 years of experience in translating manuscripts related to the academic field. The quality of the translation was considered in relation to the coincidence with the original version.

After the questionnaire was obtained, various departments belonging to the university were contacted, informed of the objective of the research, and asked for their collaboration. Students were required to obtain authorization for their participation following the recommendations of the American Psychological Association. Before the scale was administered to all participants, it was completed by a small group of people to ensure that all items were correctly understood. The administration of the scale was carried out by insisting on anonymity and that there were no true or false answers, and participants were simply asked about their own thoughts. The time to complete the questionnaire was about 10 min.

Ethical approval was obtained from the Research Ethics Committee of the University of Almeria, Spain (ref. UALBIO 2019/014).

### 2.3. Measurements

The ARS is detailed in the procedural section. The ARS of Cassidy [4] was adapted to the Spanish context. This questionnaire consisted of 30 items distributed among three factors: perseverance, 14 items; reflection and adaptation of the search for help, 9 items; negative affect and emotional response, 7 items. The following paragraph preceded all items:

You have recently received a bad grade. The grades of two other recent jobs were also lower than you would have liked since you are trying to get a good grade in the subject and therefore in the career because you have clear professional objectives and do not want to disappoint your family. The tutor’s feedback on the assignment was quite critical, including the reference to “lack of understanding” and “poor writing and expression”, but also included ways in which the work could be improved. Similar comments were made by the tutors who marked their two other assignments.

Students were required to indicate their response by means of a Likert scale from 1 (Probable) to 5 (Unlikely). As in the original scale, the score of the items with positive statements was reversed, so that a high ARS score indicated greater academic resistance.

Academic stress. The Spanish version of [30] from the student stress inventory stress manifestations [31] was used. The scale was composed of three factors: behavioral (six items), physiological (six items), and emotional (ten items). The scale was of the Likert type ranging from not at all (1) to totally agree (5).

### 2.4. Data Analysis

To determine the validity and reliability of the ARS, its psychometric properties were analyzed. First, exploratory-factor analysis (EFA) was performed; then, confirmatory-factor analysis (CFA) was performed to test its factor structure. In addition, descriptive statistical analyses were performed, and the reliability of the instrument was tested through internal consistency analysis (Cronbach’s alpha). Next, temporal-stability (intraclass correlation, ICC) and multigroup analyses to analyze gender invariance were carried out. Finally, a structural equation model (SEM) was built to analyze predictive validity. Statistical packages SPSS 25.0 (IBM, Armonk, NY, USA) and AMOS 20.0 (IBM, Armonk, NY, USA) were used for data analysis.

Because the Mardia coefficient was found to be high (183.89) for the CFA, the maximal-likelihood-estimation method was used along with a bootstrapping procedure. The estimators were not affected by lack of normality, and were therefore considered robust [32]. In order to accept or reject the tested model, several adjustment indices were considered: *χ*^2^/df, comparative fit index (CFI), incremental fit index (IFI), root mean square error of approximation (RMSEA) and its confidence interval (CI) at 90%, and standardized root mean square residual (SRMR). Values of *χ*^2^/df less than 3, values for the incremental fit index (CFI and IFI) close to or above 0.95, and values of RMSEA and SRMR less than or very close to 0.06 and 0.08 were considered, respectively, to be indicative of an adequate fit of the model to the data [33].

## 3. Results

### 3.1. Exploratory Factorial Analysis

Table 1 shows the correlation between each item and the total score of the scale, which was in the range of 0.67 and 0.83. These results led to keeping all items since the item-test correlation was higher than the cut-off point set at 0.30 [34]. Cronbach’s global alpha value was 0.82. In addition, exploratory-factor analysis supported the existence of two factors, showing a saturation factor ranging from 0.61 to 0.80.

### 3.2. Confirmatory Factorial Analysis

**Figure 1:**
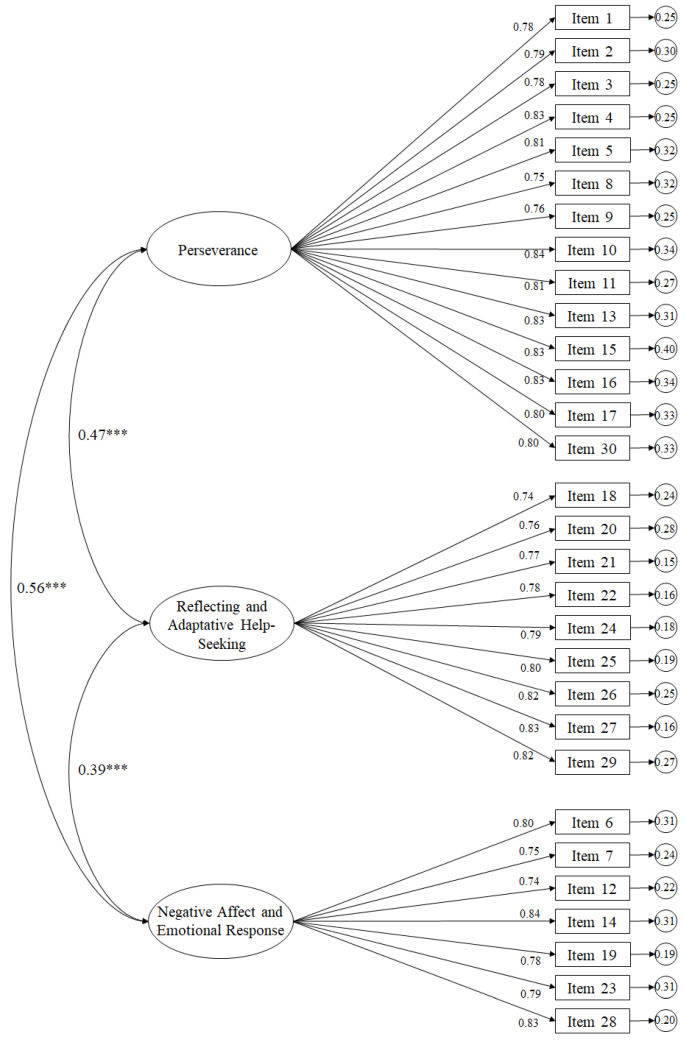
Confirmatory factorial analyses of Spanish Academic Resilience Scale. *** *p* < 0.001.

Fit rates of the tested model (Figure 1) revealed appropriate fit rates: *χ*^2^ (402. N = 723) = 1236.06, *p* < 0.001; *χ*^2^/df = 3.07; CFI = 0.96; IFI = 0.96; RMSEA = 0.056 (CI 90% = 0.053–0.068); SRMR = 0.043. Standardized regression weights ranged from 0.74 to 0.84 and were statistically significant (*p* < 0.001). Correlation between factors was statistically significant (*p* < 0.001).

Confirmatory-factor analysis of the higher-order factor was performed with acceptable adjustment rates *χ*^2^ (402. N = 723) = 996.96, *p* < 0.001; *χ*^2^/df = 2.48; CFI = 0.97; TLI = 0.97; IFI = 0.97; RMSEA = 0.049 (CI 90% = 0.040–0.055); SRMR = 0.033. There was correlation between the higher-order factor, called academic resilience, with respect to perseverance (0.59), reflex and adaptation of seeking help (0.42), and for negative affect and emotional response (0.61), all of which were statistically significant (*p* < 0.001).

### 3.3. Descriptive and Reliable Statistical Analysis

The heterotrait–monotrait ratio (HTMT) of latent factor correlations (Table 1) was 0.45, 0.51, and 0.34, suggesting the existence of discriminant validity. In addition, Table 2 shows the positive correlation between factors, highlighting the clear reciprocity between them.

Analysis of internal consistency through Cronbach’s alpha test was satisfactory, 0.88 for perseverance, 0.83 for reflection and adaptation of the search for help, and 0.86 for negative affect and emotional response.

### 3.4. Gender-Invariance Analysis

To check whether the factor structure of the model was invariant to gender, multigroup analysis was carried out. According to Table 3, no significant differences were found between (unrestricted) Model 1 and (invariance in measurement weights) Model 2. On the other hand, results did show significant differences between Models 1 and 3 (structural-invariant-covariance), and 4 (residual invariant measurement).

In the higher-order model, no significant differences were found between Models 1 (unrestricted), 2 (invariance in measurement weights), 3 (invariant structural weights), and 4 (invariant structural covariance). Results showed significant differences between Models 1, 5, (invariant structural residue) and 6 (invariant measurement residue).

The absence of significant differences between Models 1 and 2 was a minimal criterion for accepting that the model structure was invariant with respect to gender [35,36].

### 3.5. Temporary-Stability Analysis

Temporal-stability analysis, intraclass correlation coefficients (ICCs), and their confidence intervals (CI) were calculated, giving a score of 0.85 (CI = 0.82–0.90) for perseverance, 0.83 (CI = 0.80–0.88) for reflection and adaptation of seeking help, and 0.88 (CI = 0.86–0.91) for negative affect and emotional response.

### 3.6. Criterion-Validity Analysis

**Figure 2:**
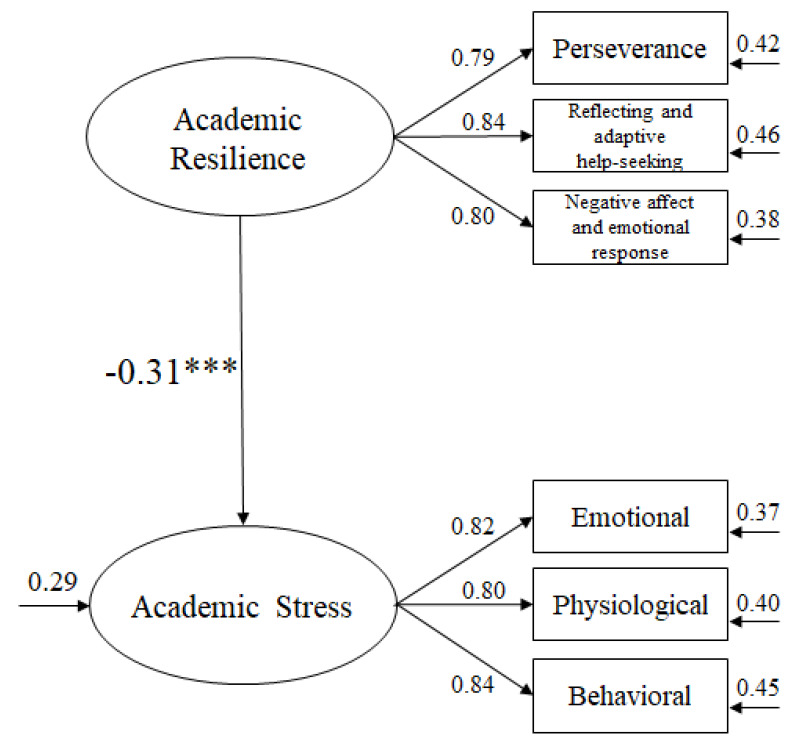
Structural equation model. *** *p* < 0.001.

To analyze the predictive validity of the scale, a SEM was carried out (Figure 2). Adjustment indices were as follows: *χ*^2^ (8. N = 1248) = 24.92, *p* < 0.001; *χ*^2^/df = 3.11; CFI = 0.97; IFI = 0.97; RMSEA = 0.052 (CI 90% = 0.045–0.060); SRMR = 0.041. According to Hair et al., the result fit established parameters.

## 4. Discussion

This study aimed to adapt and validate the ARS by Cassidy [4] to the Spanish context (Appendix A). Results achieved through factor and reliability analyses showed that the ARS is a questionnaire with adequate validity and reliability to measure academic resilience in the Spanish context. This instrument should allow for the better understanding of adolescents’ coping strategies in the face of negative experiences, making them less vulnerable to the negative effects of stressful events. To this end, it is necessary to develop and validate instruments that enable in-depth analysis of moderating variables that facilitate the development of positive responses to situations of stress and adversity.

EFA and CFA results were shown to be in line with those of the original scale of Cassidy [4], revealing the existence of three factors and supporting its factor structure. In addition, the present study showed evidence of factorial validity of higher-order-factor academic resilience, since the original questionnaire only showed evidence of reliability through Cronbach’s alpha. In this way, future studies can incorporate the global academic resilience factor into their predictive models, simplifying the complexity of studies, especially those in which the number of participants is small [36]. Invariance analyses showed that the factor structure of the scale was invariant with respect to gender that would allow for comparative analyses between men and women in future studies. Analyses of internal consistency and temporal stability were satisfactory because scores were higher than 0.80.

Finally, criterion-validity analysis through an SEM showed that academic resilience was a negative predictor of academic stress. These results were consistent with several studies in the field of social and educational psychology. In this regard, a study conducted at several Australian universities showed that students with high levels of resilience showed greater ability to adapt to academic difficulties, and were less affected by academic stress and examination pressure [37]. In the same way, a study conducted by Lee, Kang, and Kim [38] with high-school students showed that resilience negatively predicted stress and anxiety. A similar study carried out with secondary-school teachers showed that those teachers who had a great capacity to adapt to possible vicissitudes presented to them during their work performance showed a positive emotional state, lower stress and anxiety levels, and a greater capacity to recognize and regulate their own emotions [39]. Similarly, a study by Trigueros et al. [40] with a sample of semiprofessional athletes showed that those athletes who had high levels of resilience showed low levels of anxiety. Thus, results could explain the high levels of resilience associated with the generation of a series of adaptive behaviors that favor individual performance, since subjects experience higher levels of positive functioning and personal adjustment, allowing them to face potential problems with determination [41].

Despite the obtained results, it is necessary to comment on the limitations of this study. First, the nonprobability sampling method suggested that future research should analyze the factor structure of questionnaires in populations with different characteristics. Second, the study had low sample variability since subjects belonged to universities in a specific geographical area of the country. Third, future studies should analyze how certain variables (e.g., having a job, practicing sports or physical exercise, and having people under your care) can influence the academic resilience of university students, since it is living experiences and overcoming vicissitudes that influences the development of resilience [42]. Lastly, to validate the predictive capacity of the scale, it is necessary for future studies to analyze scale predictability with respect to other factors (e.g., motivation and test anxiety).

## 5. Conclusions

The results of this study support the Spanish version of the ARS (Appendix A) as a valid and reliable instrument to measure academic resilience in the Spanish context. Furthermore, the scale was shown to be invariant to gender and with adequate levels of temporal stability. On the other hand, resilience was shown to be a protective variable in the face of stressful situations that students face during their university academic period.

## Figures and Tables

**Table 1:** Correlations between each item and total scale score.

Items	Item-Test Correlation	Cronbach’s Alpha If Item is Removed	Saturation Factor of Each Item with Its Factor
1	0.67**	0.83	0.68 F1
2	0.76**	0.83	0.72 F1
3	0.75**	0.83	0.75 F1
4	0.70**	0.82	0.80 F1
5	0.80**	0.83	0.69 F1
6	0.81**	0.83	0.72 F3
	0.75**	0.82	0.73 F3
8	0.68**	0.83	0.67 F1
9	0.72**	0.83	0.72 F1
10	0.69**	0.83	0.69 F1
11	0.80**	0.83	0.63 F1
12	0.81**	0.82	0.75 F3
13	0.74**	0.83	0.72 F1
14	0.79**	0.83	0.80 F3
15	0.81**	0.83	0.70 F1
16	0.83**	0.83	0.69 F1
17	0.75**	0.82	0.64 F1
18	0.73**	0.83	0.61 F2
19	0.69**	0.83	0.73 F3
20	0.67**	0.82	0.69 F2
21	0.71**	0.83	0.65 F2
22	0.68**	0.83	0.71 F2
23	0.80**	0.83	0.68 F3
24	0.81**	0.83	0.62 F2
25	0.73**	0.83	0.64 F2
26	0.83**	0.82	0.68 F2
27	0.72**	0.82	0.72 F2
28	0.75**	0.83	0.73 F3
29	0.70**	0.83	0.75 F2
30	0.82**	0.82	0.68 F1

Note: F1 = perseverance; F2 = reflection and adaptation of the search for help; F3 = negative affect and emotional response; ** *p* < 0.01.

**Table 2:** Mean, standard deviation, Pearson correlations, and heterotrait–monotrait ratio (HTMT) proportion of factors.

Factors	*M*	*SD*	Range	AVE	1	2	3
1. Negative affect and emotional response	3.78	1.11	1–5	0.64		0.67***	0.42***
2. Perseverance	3.21	1.07	1–5	0.62	0.45		0.53***
3. Reflection and adaptation of the search for help	3.89	1.76	1–5	0.62	0.51	0.34	

Note: values below diagonal correspond to HTMT between factors; AVE = average variance extracted; *** *p* < 0.001.

**Table 3:** Gender-invariance analysis.

**Three-Factor Model**									
**Models**	***χ*^2^**	***df***	***χ*^2^/*df***	**Δ*χ*^2^**	**Δ*df***	**CFI**	**IFI**	**RMSEA (CI 90%)**	**SRMR**
1	2275.32	804	2.83			0.97	0.97	0.052 (0.046–0.057)	0.046
2	2476.38	831	2.98	28.82	27	0.96	0.96	0.053 (0.048–0.060)	0.048
3	2705.04	867	3.12	89.11***	63	0.96	0.96	0.055 (0.049–0.062)	0.052
4	2969.07	897	3.31	120.39***	93	0.95	0.95	0.056 (0.050–0.063)	0.055
**Higher-Order Model**									
**Models**	***χ*^2^**	***df***	***χ*^2^/*df***	**Δ*χ*^2^**	**Δ*df***	**CFI**	**IFI**	**RMSEA (CI 90%)**	**SRMR**
1	2275.32	804	2.83			0.97	0.97	0.052 (0.046–0.057)	0.046
2	2476.38	831	2.98	28.82	27	0.96	0.96	0.053 (0.048–0.060)	0.048
3	2597.63	863	3.01	61.49	59	0.96	0.96	0.053 (0.048–0.060)	0.049
4	2664.18	864	3.08	73.26	60	0.96	0.96	0.054 (0.048–0.061)	0.051
5	2705.04	867	3.12	89.11**	63	0.96	0.96	0.055 (0.049–0.062)	0.052
6	2969.07	897	3.31	120.39***	93	0.95	0.95	0.056 (0.050–0.063)	0.055

Note: CFI = comparative fit index; IFI = incremental fit index; RMSEA = root mean square error of approximation; CI = confidence interval; SRMR = standardized root mean square residual; ** *p* < 0.01; *** *p* < 0.001.

## Appendix A

This scale was validated in Spanish:

1. No aceptaría la opinión del tutor
2. Usaría el feedback para mejorar mi trabajo
3. Me daría por vencido
4. Usaría la situación para motivarme
5. Cambiaría de carrera
6. Probablemente me molestaría
7. Empezaría a pensar que mis posibilidades de éxito en la universidad son escasas
8. Vería la situación como un desafío
9. Haría todo lo posible para dejar de tener pensamientos negativos
10. Vería la situación como temporal
11. Trabajaría más duro
12. Probablemente me deprimiría
13. Trataría de pensar en nuevas soluciones
14. Estaría muy decepcionado
15. Yo culparía al tutor
16. Seguiría intentándolo
17. No cambiaría mis objetivos y ambiciones a largo plazo
18. Usaría mis éxitos pasados para ayudar a motivarme
19. Empezaría a pensar que mis posibilidades de conseguir el trabajo que quiero son pobres
20. Comenzaría a monitorear y evaluar mis logros y esfuerzos
21. Buscaría la ayuda de mis tutores
22. Me animaría a mí mismo
23. Evitaría entrar en pánico.
24. Probaría diferentes formas de estudiar
25. Me fijaría mis propias metas para el logro
26. Buscaría el apoyo de mi familia y amigos
27. Trataría de pensar más en mis fortalezas y debilidades para ayudarme a trabajar mejor
28. Sentiría como si todo estuviera perdido y fuera a salir mal.
29. Empezaría a autoimponerme recompensas y castigos dependiendo de mi desempeño
30. Me gustaría demostrar que puedo mejorar mis notas
